# Symptomatology and knowledge regarding pelvic floor dysfunctions and influence of gender stereotypes in female athletes

**DOI:** 10.1038/s41598-024-61464-x

**Published:** 2024-05-14

**Authors:** Elisa Bosch-Donate, Elena Vico-Moreno, Juan Carlos Fernández-Domínguez, Antonio González-Trujillo, Andreu Sastre-Munar, Natalia Romero-Franco

**Affiliations:** 1 Nursing and Physiotherapy Department, University of the Balearic Islands, Crta de Valldemossa, Km 7.5, 07122 Palma de Mallorca, Spain; 2 Health Research Institute of the Balearic Islands (IdISBa), Palma de Mallorca, Spain

**Keywords:** Pelvic floor disorders, Athletes, Female, Knowledge, Patient education, Sexual dysfunction, Urinary incontinence

## Abstract

Pelvic floor dysfunctions (PFD) are highly prevalent among females who do athletics, a sport requiring jumping, strength, and running. Although educational approaches are useful options, the educational need for this particular population remains unknown. The objective of the present study was to describe the level of knowledge regarding PFD and its relationship with symptomatology and gender stereotypes in female athletes in Spain. A total of 255 female athletes completed an anonymous online survey to explore their knowledge regarding urinary incontinence (UI), pelvic organ prolapse (POP), anal incontinence (AI), and sexual dysfunction (SexD), as well as their PFD symptoms and gender stereotyped beliefs related to sport. Educational level and sports characteristics (training volume, experience, and athletic modality) were also explored. Participants demonstrated a low level of knowledge in terms of POP (52.5%), AI (64.0%), and SexD (40%), but not for UI (70.8%). The proportion of PFD complaints was 63.5% for dyspareunia, 51.8% for urine leakage, 42.4% for pelvic pain, 17.3% for AI, and 9.0% for POP, with no associations with knowledge (*p* > 0.05). Lower knowledge about UI and SexD was related to greater gender stereotypes (*p* < 0.05) and rejection of professional healthcare (*p* = 0.010). As a conclusion, the level of knowledge about PFD was low in female athletes who train and compete in athletics in Spain, mainly with regard to sexual dysfunction. Although 63.5% of athletes had dyspareunia and 51.8% urinary leakages, symptomatology was not associated with level of knowledge. However, a lower level of knowledge was associated with more stereotyped beliefs and rejection of professional healthcare for PFD. These findings confirm the need to design appropriate educational interventions to disseminate information on all the types of PFD, particularly sexual contents. The potential influence of gender stereotypes makes it appropriate to include the gender perspective in these interventions.

## Introduction

Pelvic floor dysfunctions (PFD) are highly prevalent among female athletes when comparing with males or with non-athlete females^1,2^. This prevalence is even higher in so-called impact sports, such as most athletics modalities^3^. For optimal sports performance in these modalities, athletes require jumping, strength, and/or running. These tasks increase intra-abdominal pressure and stress in pelvic floor structures^4,5^.

As conservative options to prevent and manage PFD, educational approaches are one of the first line conservative options for the general population^6^. Although female athletes often have greater body awareness and healthcare information compared to a sedentary population, silence, normalization, and embarrassment regarding the pelvic floor (PF) may reduce requests for healthcare and self-care consultations to ensure PF health^7^. In this vein, no studies to date have explored the knowledge of PFD in a sports population that is especially affected by PFD, such as female athletes who train and compete in athletics^2^. Thus, the existing educational needs for this type of population remains unknown. 

Meanwhile, gender stereotypes are socially and culturally learned behaviors, with a strong influence in the sport context^8^. Consequently, gender stereotypes contribute to girls and women being physically less active than men^9^, often having fewer opportunities for participation in sports, obtaining less recognition from sport, or achieving less successful sports careers^10^. Despite these inequalties^11^, disorders that are more prevalent in female compared to male athletes, such as PFD, have been approached by focusing only on biological sex determinants^12^. This management avoids exploring the influence of sociocultural determinants^12^. Previous studies have demonstrated that gender stereotypes may affect self-image and self-care of athletes^13^. This potential impact could play a significant role in the normalization of disorders that are especially prevalent among female athletes, such as PFD. The consequent low knowledge and lack of seeking professional healthcare related to PFD could perpetuate or worsen their symptomatology. To the best of our knowledge, no studies have yet explored the influence of gender stereotypes on healthcare and knowledge regarding PFD in a sports population to date. Thus, the potential influence of gender stereotypes among female athletes needs exploring, in order to detect and break these beliefs.

Based on these arguments, this study aimed to describe the level of knowledge regarding the different types of PFD and its relationship with PFD symptomatology and gender stereotypes in female athletes who do athletics in Spain. We hypothesized that athletes have a low level of knowledge of PFD, mainly concerning contents related to sexual, anal, and prolapse dysfunctions.

## Materials and methods

### Design

A cross-sectional observational study was designed. During May and June, 2023, female athletes who did athletics in Spain completed an anonymous online questionnaire to evaluate their knowledge of PFD. Socio-demographic, sports-related, occurrence of PFD symptoms, and other medical information related to pelvic floor function, as well as beliefs related to gender stereotypes in sport were collected. All methods were performed in accordance with the CROSS guidelines^14^.

### Participants

All female athletes with a sports license for athletics in Spain were invited to participate via e-mail from their regional federations, clubs, and/or training groups. By way of inclusion criteria, female athletes had to train and compete for any athletic modality and be at least 18 years old.

At the time of the study, the National Sports Council certified 41,400 athletics licenses for female athletes, 31,000 of these for female athletes at least 18 years of age. In this term, it is needed to consider that the maximal proportion of female athletes with PFD is 50%, according to previous studies^2^. Thus, for sample size calculation, a finite population proportion was considered, with 95% confidence level and 7% accuracy in the study, requiring a minimum sample size of 196 athletes. The sample size was estimated through the web-based application GRANMO (Barcelona, Spain, version 7.04).

Only female athletes who did not have the ability to adequately understand questions in Spanish, as the national language, were excluded. A total of 255 athletes agreed to participate in the study and completed the online questionnaire. Prior to filling in the questionnaire, all participants were informed of the objectives and procedures of the study and gave their informed consent to participate in the study through an electronic survey tool (JotForm®, San Francisco, USA). All data were anonymous and confidential in line with new continental data protection and national norms. This study was approved by the Ethical Committee of the University of the Balearic Islands (ref: 124CER19) and was conducted according to the Code of Ethics of the World Medical Association (Declaration of Helsinki).

### Procedures

Online questionnaire: Participants were asked to complete an anonymous online questionnaire through the Jotform® platform (San Francisco, USA). The questionnaire consisted of the following sections: 1) Socio-demographic data: age and academic studies; 2) Sports-related information: athletic modality, experience (years), and weekly training volume (hours/week); 3) Medical information related to occurrence of PFD, based on questions extracted from previously validated tools (as recommended)^15^: urinary leakage and impact on their daily life (0—none to 10—maximal) (extracted from the ICIQ—International Consultation on Incontinence Questionnaire^16^, pelvic organ prolapse (POP) (extracted from the P-QoL—Prolapse Quality of Life Questionnaire^17^, anal incontinence (AI) and type of component (gas, mucous, liquid and solid stools) (extracted from the Wexner scale^18^, dyspareunia (extracted from the FSFI—Female Sexual Function Index^19^, and pelvic pain (question based on the chronic pelvic pain definition from Moore and Kennedy^20^, previous labors and PFD healthcare seeking behavior; 4) Beliefs regarding gender stereotypes in sport: the 1^st^ (F1GS) and 3^rd^ factor (F3GS) from the validated CEGAFD questionnaire (*Creencias y Estereotipos de Género relacionados con la Actividad Física y el Deporte*) were selected^21^; 5) Knowledge regarding UI (Urinary Incontinence): 12 items extracted from the PIKD-UI (Prolapse and Incontinence Knowledge Questionnaire-Urinary Incontinence); 6) Knowledge related to POP: eight items selected from the PIKD-POP (Prolapse and Incontinence Knowledge Questionnaire-Pelvic Organ Prolapse) (both PIKD-UI and PIKD-POP subscales have been validated in Spanish^22^; 7) Knowledge regarding AI: 10 ad-hoc items; 8) Knowledge regarding sexual dysfunction: 10 ad-hoc items. Due to the absence of existing items related to AI and sexual dysfunction in athletes, the research team developed new items, similarly to previous studies^23^. The knowledge Sects. (5^th^ to 8^th^) presented questions with three answers: “true”, “false”, and “I do not know”. Like previous studies, a score of 1 was recorded for each correct response, a score of 0 for incorrect responses or “I do not know”^24^. The sum and percentage of individual correct responses were specifically calculated for each knowledge section (UI, POP, AI, and sexual dysfunction). Only one member of the research team had access to this data with unique logins, thus preventing unauthorized access. To prevent “multiple participation” of athletes, the option “Unique submission" was selected during the questionnaire design on the JotForm platform. This option prevented multiple submission from the same browser/IP address. To reduce non-response bias, several strategies were used: the questionnaire was pretested with potential respondents; the purpose of the questionnaire, approximate time to complete it, and information about anonymity and confidentiality of data were stated at the beginning; athletes were advised, just before submitting the questionnaire, that the message “we have received your response” should appear. Since no contact information was collected in order to maintain anonymity and confidentiality of participants, the option of “save and continue later” was not available. All items of the questionnaire are shown in Supplementary File 1. 

### Statistical analysis

Before the start of the study, face and content validity of the questionnaire were verified for ad-hoc sections of the questionnaire (7^th^ and 8^th^ sections, on anal and sexual dysfunctions, respectively). To this end, collaboration of three experts (three pelvic floor therapists), and one independent expert (a general practitioner) was requested. Additionally, 15 athlete volunteers completed the questionnaire and confirmed the appropriateness of the aforementioned items and the time needed to complete it^25^. For validity, open field options were included in each section to allow suggestions or comments related to adequacy and relevance criteria (content validity), as well as understanding and clarity (face validity). Internal consistency of ad-hoc Sects. (7th to 8th) was measured via Cronbach’s alpha coefficient. The cut-off for Cronbach’s alpha coefficient is 0.7 as acceptable, 0.8 as good, and ≥ 0.9 as excellent^26^.

Descriptive analyses were conducted using mean and standard deviations (SD) for numerical variables, and frequencies or percentages for categorical variables. Some quantitative variables (age) were transformed into qualitative variables through division by the median, as in previous studies^24^. The association of knowledge was determined with variables related to sport (athletic experience and training volume) and gender stereotypes through the Pearson correlation test; while the association of knowledge with socio-demographic (age, educational level, health-related education), sports- (athletic modality), and health-related (health care demands, previous labors, PFD symptomatology) variables was ascertained through analysis of variance (ANOVA). The Bonferroni post hoc test was used to evaluate between-group differences. Confidence intervals (CI) 95% were calculated for all differences and Cohen’s d effect sizes (ES) were calculated to determine the magnitude of the differences between groups, interpreted as small (d = 0.2), medium (d = 0.5), large (d = 0.8), or very large (d = 1.3)^27^. The Statistical Package for Social Science (SPSS) was used for statistical analysis (Chicago, IL, USA, version 24.0). An alpha level of 0.05 was used to indicate statistical significance.

## Results

In terms of content validity, the three experts indicated that the questions were properly interpreted, measured what they were intended to, and that the content assessed all fundamental aspects of PFD. For the face validity, 15 female athletes completed the questionnaire, for an actual response rate of 100%. Only minor changes in the writing of the items were made to improve clarity and understanding. The time required to complete the questionnaire ranged from 12 to 20 min. Adequate internal consistency was confirmed for all knowledge subsections (7th to 8th), with Cronbach’s alpha values of 0.70 for the 7th section (knowledge regarding anal dysfunction) and 0.75 for the 8^th^ section (knowledge regarding sexual dysfunction).

During the period of the study, 255 female athletes (35.2 ± 11.6 years) completed the online questionnaire. Table 1 shows the baseline characteristics and those related to academic and sports experience of all female athletes, as well as their score in terms of gender stereotypes in sport.

**Table 1:** Characteristics of all female athletes.

	All participants (n = 255)
Age (%)	
Under 35 years	52.3
Over 35 years	47.7
Level of academic studies (%)	
Elementary	0.8
Secondary	9.4
Medium or high grade	12.5
University degree	38.4
Master’s degree	34.5
PhD	4.3
Health-related studies (%)	
Yes	49.8
No	50.2
Athletics modality (%)	
Sprint and hurdles	15.2
Middle-distance running	12.3
Long-distance running	34.3
Horizontal jumping	3.6
Vertical jumping	2.2
Throwing	3.6
Racewalking	2.2
Combined events	4.0
Trail	14.8
Athletics experience (yrs)^¥^	13.5 (9.3)
Weekly volume training (h/week)^¥^	8.6 (5.0)
Weekly volume training (sessions/week)^¥^	5.0 (1.7)
Previous labors (%)	
Multiparous	38.0
Nulliparous	62.0
PFD professional healthcare (%)	
Rejected	12.2
Accepted	87.8
Differences associated with gender, related to sport (Factor 1—CEGAFD)^¥^	13.4 (4.1)
Presence of gender stereotypes related to sport (Factor 3—CEGAFD)^¥^	9.9 (3.7)

CEGAFD, beliefs and gender-based stereotypes concerning physical activity and sport; PFD, pelvic floor dysfunctions; ^¥^Data are given as Mean (standard deviation).

Occurrence of PFD symptoms are shown in Table 2. The highest proportion of PFD complaints were related to painful sexual relationships (63.5%) and urine leakages (51.8%). Females who claimed to have urine leakage scored 3.9 ± 2.8 points (95% CI 3.5–4.4) (with 10 points as the maximum score) as the average impact on their quality of life due to these urine leakages. The occurrence of AI and POP was 15.8 and 9.0%, respectively, with loss of gas as the main component of anal incontinence (65.9% loss of gas, 22.5% loss of liquid stool, 11.6% loss of solid stool).

**Table 2:** Symptoms related to pelvic floor of all female athletes.

Do you have urinary leakage?^a^ (%)		Do you have anal leakage?^b^ (%)		Do you feel pain during sexual relationships?^c^ (%)(%)		Do you have a sensation that there is a bulge in your vagina or that something is falling out of your vagina?^e^ (%)		During the last three months, have you had chronic or persistent pain inside your pelvis?^d^	
No, never	48.2	No, never:	82.7	No, never	36.5	No, never	57.6	No, never	91.0
Yes:	51.8	Yes:	17.3	Yes:	63.5	Yes:	42.4	Yes:	9.0
Once a week or less	33.3	Once a week or less	10.6	Rarely	34.1	Rarely	20.8		
Two or three times a week	10.6	Two or three times a week	2.0	Sometimes	22.7	Sometimes	14.1		
Once a day	3.1	Once a day	1.6	Frequently	4.7	Frequently	5.5		
Two or three times a day	3.9	Two or three times a day	2.4	Always	2.0	Always	2.0		
Continuously	0.8	Continuously	0.8						

^a^Extracted from ICIQ (International Consultation on Incontinence Questionnaire); ^b^Extracted from FISI (Anal incontinence Severity Index); ^c^Extracted from PISQ-IR (Pelvic organ prolapse incontinence sexual questionnaire – IUGA revised); ^d^Ad-hoc question, based on pelvic chronic pain definition, from Moore and Kennedy, 2000^20^; ^e^extracted from EPIQ (epidemiology of prolapse and incontinence questionnaire).

Table 3 shows the scores of knowledge regarding PFD (UI, POP, AI, and SexD). The lowest score was observed in knowledge of SexD, with 4.0 correct responses (95% CI 3.7–4.3) out of a possible 10 (40.0% as the average of individual correct responses). The highest score was obtained for UI, with 8.5 correct responses (95% CI 8.1–8.8) out of 12 possible ones (70.8% as the average of individual correct responses).

**Table 3:** Knowledge about pelvic floor dysfunctions of all athletes according to the characteristics.

	UI Knowledge (correct responses)			POP Knowledge (correct responses)			AI knowledge (correct responses)			Sexual knowledge (correct responses)		
	Mean (n, %)	(95% CI) (%)	*p*-value	Mean (n, %)	(95% CI) (%)	*p*-value	Mean (n, %)	(95% CI) (%)	*p*-value	Mean (n, %)	(95% CI) (%)	*p*-value
All athletes	8.5, 70.8	(67.5 – 73.3)	n/a	4.2, 52.5	(48.8 – 56.3)	n/a	6.4, 64.0	(61.5 – 66.5)	n/a	4.0, 40.0	(37.2 – 43.1)	n/a
Age			0.358			0.083			0.190			0.125
Under 35 years	8.3, 69.3	(64.7–73.5)		4.0, 49.8	(44.9–54.8)		6.2, 62.3	(58.4–66.0)		4.2, 42.2	(38.2–46.6)	
35 years and over	8.6, 72.0	(68.7–75.3)		4.5, 55.8	(51.4–60.3)		6.6, 65.9	(62.4–69.4)		3.8, 37.6	(33.7–41.6)	
Academic studies			0.459			0.770			0.561			0.031
Elementary	6.0, 50.0	(30.0–90.0)		2.5, 31.3	(10.0–40.0)		6.0, 60.0	(40.0 – 80.0)		1.5, 15.0	(5.0–30.0)*	
Secondary	7.7, 63.9	(52.6–74.0)		4.5, 56.3	(43.2–68.5)		6.5, 64.6	(56.4–71.8)		3.3, 32.5	(24.2–40.4)	
Medium or high grade	8.6, 71.9	(66.1–77.0)		4.0, 50.0	(42.2–57.6)		6.4, 63.8	(55.0–71.0)		3.7, 37.2	(30.4–43.7)	
University degree	8.5, 70.9	(65.3–74.7)		4.2, 53.1	(47.7–58.5)		6.5, 64.8	(60.2–69.4)		4.0, 39.9	(35.1–44.7)	
Master’s degree	8.7, 72.8	(68.1–77.9)		3.7, 53.6	(47.6–59.7)		6.5, 64.8	(60.9–68.9)		4.5, 45.2	(40.4–50.4)	
PhD	8.5, 71.2	(56.5–83.3)		3.7, 46.6	(29.8–63.9)		5.2, 51.8	(36.5–64.5)		2.8, 28.2	(15.0–43.6)	
Health-related studies			< 0.001			0.015			< 0.001			< 0.001
No	7.8, 64.9	(61.0–68.6)		3.9, 48.5	(43.7–53.3)		5.9, 58.5	(54.8–62.2)		3.4, 33.5	(29.8–37.2)	
Yes	9.2, 76.3	(72.4–80.2)***		4.6, 56.9	(52.1–61.5)***		7.0, 69.6	(66.3–72.8)***		4.7, 46.5	(42.6–50.7)***	
Athletic modality			0.064			0.065			0.026			0.071
Sprint and hurdles	7.4, 61.5	(53.5–68.6)		3.4, 42.6	(34.1–50.9)		5.7, 57.4	(49.8–64.3)		3.1, 31.4	(25.0–38.0)	
Middle-distance running	8.0, 66.7	(58.3–74.5)		4.6, 57.0	(49.5–64.5)		6.8, 68.2	(62.6–74.1)*		4.3, 43.2	(35.3–51.5)	
Long-distance running	9.1, 75.6	(71.8–79.6)		4.6, 57.4	(51.8–63.0)		6.8, 68.4	(64.6–72.3)		4.5, 44.8	(40.4- 49.8)	
Horizontal jumping	8.7, 72.5	(60.7–83.3)		3.0, 37.5	(20.8–56.2)		5.4, 54.0	(43.8–65.0)		3.0, 30.0	(16.7–47.0)	
Vertical jumping	9.5, 79.2	(68.3–91.7)		4.7, 58.3	(37.5–77.5)		6.3, 63.3	(40.0–84.0)		4.2, 41.7	(20.0–61.2)	
Throwing	8.8, 73.3	(61.1–81.7)		3.9, 48.8	(30.6–65.6)		6.1, 61.0	(51.1–72.0)		3.5, 35.0	(21.7–47.8)	
Racewalking	8.3, 69.4	(36.6–87.5)		3.2, 39.6	(18.8–62.5)		5.3, 53.3	(27.4–77.5)		3.2, 31.7	(15.0–48.1)	
Combined events	7.5, 62.9	(46.3–78.8)		4.0, 50.0	(35.7–66.7)		5.2, 51.8	(35.0–70.0)		3.3, 32.7	(17.5–50.0)	
Trail	8.6, 71.3	(64.2–78.4)		4.4, 55.2	(45.8–64.3)		6.5, 65.4	(58.7–72.0)		4.2, 41.5	(34.1–49.0)	
Previous labors			0.949			0.078			0.281			0.091
No	8.5, 70.5	(66.6–74.2)		4.0, 50.3	(45.8–54.9)		6.3, 62.9	(59.4–66.3)		4.2, 42.0	(38.4–45.6)	
Yes	8.5, 70.7	(66.5–74.6)		4.5, 56.6	(51.6–61.5)		6.6, 65.9	(62.2–69.7)		3.7, 36.8	(32.3–41.2)	
PFD healthcare			< 0.001			0.010			< 0.001			< 0.001
Rejected	6.5, 53.8	(43.2–63.3)		3.3, 40.7	(31.3–50.5)		5.1, 50.6	(41.4–58.9)		2.4, 23.5	(16.5–31.4)	
Accepted	8.7, 72.9	(70.3–75.7)***		4.4, 54.4	(50.8–57.9)***		6.6, 65.9	(63.2–68.8)***		4.2, 42.3	(39.5–45.5)***	

AI, anal incontinence; CI, confidence interval; n/a, not applicable; PFD, pelvic floor dysfunction; POP, pelvic organ prolapse; UI, urinary incontinence; Significant differences compared to the rest of groups *p < 0.05;***p < 0.001.

Comparisons of the knowledge scores according to categorical variables are also presented in Table 3. There were no significant differences between groups according to age (*p* > 0.05). Subgroups according to level of academic education were found to differ significantly (*p* = 0.031), with the athletes with only elementary level education receiving lower scores than those with a higher level of academic education. Athletes with health-related studies scored higher in all the knowledge sections. Sub-groups according to athletic modality were found to be different for knowledge of sexual dysfunction (*p* = 0.026), with higher scores for athletes specializing in long-distance events. Likewise, female athletes who rejected professional healthcare for PFD scored lower in all the knowledge sections (*p* < 0.010). There were no significant differences between groups in terms of previous labors (*p* > 0.05).

Correlations of the knowledge scores with numerical variables (athletic experience, athletic training, and score regarding gender stereotypes in sport) did not show any significant result (*p* > 0.05), except for gender stereotypes, which showed small yet significant negative correlations. Athletes who had more stereotyped beliefs scored lower values for knowledge regarding UI (F1GS: r = − 0.124, *p* = 0.048; F3GS: r = − 0.142, *p* = 0.024) and SexD (F1GS: r = r = − 0.150, *p* = 0.016; F3GS: r = − 0.142, *p* = 0.023).

Table 4 shows comparisons of the knowledge scores according to PFD symptomatology in female athletes. Significant differences were observed in the POP knowledge section, with female athletes who had POP (*p* = 0.022) receiving a higher score than the rest of athletes. No other significant differences were found (*p* > 0.05).

**Table 4:** Knowledge according to symptomatology of female athletes.

	UI knowledge (correct responses)			POP knowledge (correct responses)			AI knowledge (correct responses)			Sexual knowledge (correct responses)		
	Mean (n, %)	(95% CI) (%)	*p*-value	Mean (n, %)	(95% CI) (%)	*p*-value	Mean (n, %)	(95% CI) (%)	*p*-value	Mean (n, %)	(95% CI) (%)	*p*-value
Urinary leakage			0.780			0.797			0.610			0.561
No	8.5, 71.0	(66.7–75.1)		4.2, 52.2	(47.4–56.8)		6.3, 63.3	(59.6–66.8)		3.9, 39.1	(35.0–43.2)	
Yes	8.4, 70.2	(66.1–73.8)		4.2, 53.1	(48.4–57.9)		6.5, 64.7	(60.9–68.3)		4.1, 40.8	(36.9–44.7)	
Dyspareunia			0.678			0.287			0.520			0.956
No	8.4, 69.8	(64.9–74.3)		4.0, 50.3	(44.8–55.7)		6.3, 62.9	(58.0–67.5)		4.0, 40.1	(35.3–44.7)	
Yes	8.5, 71.0	(67.6–74.5)		4.3, 54.1	(49.8–58.2)		6.5, 64.7	(61.7–67.9)		4.0, 39.9	(36.5–43.8)	
Anal leakage			0.731			0.851			0.651			0.142
No	8.5, 70.8	(67.7–73. 9)		4.2, 52.5	(48.9–56.6)		6.4, 64.3	(61.3–67.1)		4.1, 41.0	(37.7–44.3)	
Yes	8.3, 69.5	(62.3–76.5)		4.3, 53.5	(45.7–62.2)		6.3, 62.7	(56.5–68.9)		3.5, 35.2	(27.7–42.4)	
Pelvic pain			0.811			0.060			0.393			0.780
No	8.4, 70.3	(66.6–74.2)		4.0, 49.9	(45.2–54.2)		6.3, 63.1	(59.3–66.6)		4.0, 39.5	(36.0–43.2)	
Yes	8.5, 71.0	(66.5–75.0)		4.5, 56.5	(51.3–61.4)		6.5, 65.4	(61.6–69.0)		4.1, 40.6	(35.6–45.3)	
POP			0.680			0.022			0.704			0.196
No	8.4, 70.4	(67.2–73.4)		4.1, 51.5	(47.9–54.9)		6.4, 63.9	(61.1–66.7)		4.1, 40.6	(37.5–43.8)	
Yes	8.7, 72.5	(64.6–79.3)		5.2, 65.2	(57.4–72.8)*		6.6, 65.7	(58.2–73.7)		3.4, 33.9	(24.5–43.4)	

AI, anal incontinence; CI, confidence interval; UI, urinary incontinence; POP, pelvic organ prolapse; SD, standard deviation; *Significant differences compared to the rest of groups (*p* < 0.05).

## Discussion

This study aimed to describe the level of knowledge regarding the different types of PFD and its relationship to PFD symptomatology and gender stereotypes in female athletes who do athletics in Spain. As hypothesized, our findings showed that female athletes had a low level of knowledge regarding PFD, with a score of less than 70% for all dysfunctions except for UI (70.8%). Knowledge was especially limited for the section related to SexD, with an average score of ~ 40%. Symptomatology related to PFD was highly prevalent among female athletes, with 63.5% of athletes claiming to have dyspareunia and 51.8% urinary leakages. However, no associations were found between level of knowledge regarding PFD and PFD symptomatology. As an interesting result, higher scores in stereotyped beliefs related to sport were associated with a lower level of knowledge. In this vein, female athletes who rejected PFD professional healthcare had a lower level of knowledge in all sections and higher stereotyped beliefs with regard to sport.

In line with previous studies, if we considered ≥ 70% accuracy a cut-off for adequate knowledge and < 70% for low or inadequate knowledge^24^, athletes reached an adequate level of knowledge by a slight margin only when related to UI (CI 95% 67.5–73.3%). Our results agree with previous studies that found a gap in knowledge regarding PFD among females^28^, and confirmed the same trend among athletes. We also found that a lower educational level is associated with this gap^23,28^. Although having health-related studies helps obtain a higher level of knowledge as regards all types of PFD, it did not imply reaching an adequate level of knowledge concerning POP and SexD. In our study, almost half the participants were involved in health-related studies, in line with the study by Sotoca, who reported that most of the Spanish athletes who participated in athletics in the Olympic Games (Tokyo 2020) were involved in health-related university studies^29^. When exploring athletes according to their athletic modalities, athletes specializing in middle- and long-distance running demonstrated greater knowledge of AI. Although these athletes had a score of 68%, this did not reach the adequate level of knowledge proposed by scientific literature^24^. 

When exploring the level of knowledge regarding each PFD, the lowest score was observed for SexD (~ 40%), followed by POP (~ 53%). These data are especially relevant in the normalization of this dysfunction if we consider that over 63% of athletes claimed to have dyspareunia. This result is in line with previous studies that observed an occurrence of dyspareunia in most of the athletes assessed^24^. Although knowledge regarding sexual dysfunction was not associated with the occurrence of dyspareunia in our study, it would be appropriate to design educational interventions to make athletes alert and fully aware of a potential sexual dysfunctional situation^30^. Disseminating information about treatment and prevention options could be helpful to increase healthcare requests from athletes^31^. As previous studies confirmed, having adequate knowledge may help reduce the chance of developing PFD^24^ and also help improve proprioception^31^. Although having a PFD dysfunction might be expected to motivate athletes to find information about it, this behavior did not occur for UI or SexD, similarly to previous studies^32^. Once again, the normalization of urinary leakages and dyspareunia may be the main explanation. In the case of POP, females often have the feeling “like a tampon is falling out”^33^, as well as frequently related symptomatology such as pelvic pressure, groin and lower back pain, painful intercourse, or difficult bowel movements, among others^34^. The impact of these symptoms on the quality of life makes women seek professional healthcare and information and explains the higher level of knowledge of women who have POP compared to women who do not^35^. In our study, 9.0% of athletes claimed to have a feeling of a bulge in their vagina, in line with previous studies that found a prevalence ranging from 1.4% in Cross Fit participants^36^ to 23.0% in Olympic weightlifters and power lifters^37^.

Regarding knowledge about UI, it is the most known PFD among athletes. This result is consistent with the volume of scientific literature and educational interventions focusing on UI compared to the rest of PFD, according to authors of recent reviews^15,28,38^. Once again, although level of knowledge was not associated with occurrence of urinary leakage, dissemination of information would motivate athletes to ask for professional healthcare and avoid normalizing loss of urine during training^39^. In our study, despite the high prevalence of urinary leakage among athletes (51.8%), the frequency was once per week or less in most cases (64.3% in athletes who had urinary leakage). Although this low frequency may be the main reason why athletes perceived only a slight impact on their daily life (3.8 points on a 0–10 scale), previous studies also suggest the normalization of loss of urine as a point^24^. In the same vein, other observational studies that explored UI prevalence in female athletes in Spain observed 44.4%^40^ in amateur athletes and 51.7% in elite athletes^2^.

Our results suggest a certain association between gender stereotypes in sport with low knowledge and rejection of seeking professional healthcare for PFD. Having higher stereotyped gender beliefs also seems to be associated with a lower level of knowledge and rejection of seeking professional healthcare for PFD. Although athletes would be expected to reject professional healthcare when they do not have adequate knowledge regarding PFD, it is important to consider the potential influence of gender stereotyped beliefs in the normalization of these dysfunctional situations and the design of appropriate educational strategies. To our knowledge, this is the first study to explore the existence of gender stereotypes in athletes and its relationship with knowledge and medical information regarding PFD, such as the occurrence of PFD symptomology. A recent qualitative study that explored the potential influence of cultural aspects related to sports contexts in women with PFD observed that the culture of the sporting environment was a factor that affected women’s decision to continue or cease exercise when suffering from PFD^41^. Women stated they feel uncomfortable in sports contexts that are mostly made up of male members and a masculine culture because men did not understand their situation or did not demonstrate any interest in hearing about it^41^. Our results confirm the need to encourage a supportive and inclusive culture within sports contexts.

Considering the occurrence of PFD in female athletes, our results are in line with Cardoso et al., who found complaints of dyspareunia from most athletes. In our study, 53.7% of all athletes who claimed to have dyspareunia reported “rarely” as a frequency. Cardoso et al. did not report the frequency of this occurrence. Athletes deserve to know that, even when occurring with a rare/low frequency, the existence of pain during sexual relationships is a dysfunctional situation that can be managed by health professionals. Similarly, a high occurrence of pelvic pain was observed in our study (> 42%). The lack of studies exploring pelvic pain occurrence as PFD in female athletes makes the comparison of our results^38^. Studies that explored the influence of PFD symptoms in exercise participation in women observed 21% pelvic pain prevalence, but these women were not athletes^41^. In the present study, none of the PFD were associated with sports characteristics of athletes, such as training volume, athletic modality, or sports experience, which is in line with studies exploring sports population^2^. Although some studies reported jumping as the most provocative sports practice^2^, we did not collect information regarding type of provocative exercises or time when symptoms occurred in relation to sports practice. Exploring the circumstances of urinary and anal leakages (i.e. during sports practice, outside of sports practice, or both) would be useful in order to delve into the influence of PFD occurrence in the continuation, reduction, or cessation of sports practice.

Our study had limitations. Firstly, our results are based on self-identified PF symptoms through key questions extracted from validated questionnaires. Even though the management of PFD is based on the patient's complaint and, therefore, symptomatology, exploring these symptoms by physical examination could be useful to detect additional clinical signs. Although online data collection made it possible to disseminate the questionnaire to more female athletes, selection bias could have influenced results by including females more interested in PFD than athletes who did not reply to the questionnaire. Additionally, in our study, questions were limited to the most representative points so as to avoid potential withdrawals due to the length of the questionnaire. This aspect made it difficult to categorize urinary or fecal incontinence, explore other potential risk factors such as constipation and urinary infections, or describe other factors associated with good lifestyle habits. Instead, the PIKD questionnaire was carefully selected to explore knowledge about UI and POP as an appropriate instrument that has been validated in Spanish females. Additional ad-hoc questions had to be designed to specifically address the rest of PFD. Besides, since the option “save and continue later” was not available in the survey to avoid collecting contact information of participants, only the responses from athletes who completed the entire survey and submitted it were collected, which could have limited the participation of more athletes. Finally, participants were all female athletes who trained and competed in an athletic modality in Spain, hence it would not be appropriate to extrapolate our results to another sports population. 

Our results confirmed the need to design appropriate educational interventions to disseminate information focused on all types of PFD. Due to the low level of knowledge especially observed for sexual dysfunction and the high occurrence of urinary leakage and dyspareunia, educational strategies should include information focused on SexD as well as UI, as priority points. The potential influence of gender stereotypes as cultural aspects in sports settings make it appropriate to include the gender perspective when designing these educational interventions. Further, it is also necessary to raise awareness among sports supervisors because the risk of PFD is barely considered in the training of sportswomen.

By way of conclusion, the level of knowledge regarding PFD related to almost all types of PFD was low in female athletes who train and compete in athletics in Spain. This level was especially low with regard to SexD but higher related to UI. Although 63.5% of athletes had dyspareunia and 51.8% had urinary leakages, symptomatology was not associated with level of knowledge. However, a lower level of knowledge was associated with greater stereotyped beliefs and rejection of seeking professional healthcare for PFD.

### Supplementary Information

Supplementary Information.

## Data Availability

The datasets used and analyzed during the current study are available from the corresponding author on reasonable request.
