# Supplementary material for: Symptomatology and knowledge regarding pelvic floor dysfunctions and influence of gender stereotypes in female athletes
Source: Sci Rep. 2024 May 14;14:11052. doi: 10.1038/s41598-024-61464-x (PMC11094071; doi:10.1038/s41598-024-61464-x)
Supplement: Supplementary file 1 — Supplementary Information. [file 41598_2024_61464_MOESM1_ESM.doc]

**Supplemental file 2. Knowledge questions in the online questionnaire**

**CONOCIMIENTOS SOBRE INCONTINENCIA URINARIA**

A continuación, te presentamos una serie de preguntas sobre la incontinencia urinaria, como una de las disfunciones del suelo pélvico. Por favor, contesta a cada una de las afirmaciones si estás de acuerdo, en desacuerdo o no lo sabes

1. **La incontinencia urinaria es más común en mujeres jóvenes que en mujeres mayores.**

Estoy de acuerdo Estoy en desacuerdo La verdad es que no lo sé

1. **Las disfunciones de vejiga son más frecuentes en mujeres que en hombres.**

Estoy de acuerdo Estoy en desacuerdo La verdad es que no lo sé

1. **No se puede hacer mucho para tratar las pérdidas de orina, a excepción del uso de compresas, salvaslip o pañales.**

Estoy de acuerdo Estoy en desacuerdo La verdad es que no lo sé

1. **Para el tratamiento de la pérdida de orina no es importante realizar un diagnóstico previo del tipo de pérdida.**

Estoy de acuerdo Estoy en desacuerdo La verdad es que no lo sé

1. **Las pérdidas de orina pueden deberse a múltiples causas.**

Estoy de acuerdo Estoy en desacuerdo La verdad es que no lo sé

1. **Efectivamente, los ejercicios pueden ayudarnos a controlar la pérdida de orina.**

Estoy de acuerdo Estoy en desacuerdo La verdad es que no lo sé

1. **Algunos medicamentos pueden causar la pérdida de orina.**

Estoy de acuerdo Estoy en desacuerdo La verdad es que no lo sé

1. **Una vez que la persona comienza a perder orina, nunca vuelve a ser capaz de controlarla.**

Estoy de acuerdo Estoy en desacuerdo La verdad es que no lo sé

1. **Para diagnosticar las pérdidas de orina, se pueden hacer test especiales según el tipo de disfunción.**

Estoy de acuerdo Estoy en desacuerdo La verdad es que no lo sé

1. **La cirugía es el único tratamiento para las pérdidas de orina.**

Estoy de acuerdo Estoy en desacuerdo La verdad es que no lo sé

1. **Dar a luz muchas veces puede causar pérdidas de orina.**

Estoy de acuerdo Estoy en desacuerdo La verdad es que no lo sé

1. **La mayoría de la gente que tiene pérdidas de orina puede curarse o mejorar con algún tipo de tratamiento.**

Estoy de acuerdo Estoy en desacuerdo La verdad es que no lo sé

**CONOCIMIENTOS SOBRE DISFUNCIONES SEXUALES**

A continuación, te presentamos una serie de preguntas sobre la función sexual. Por favor, contesta en cada una de las preguntas si estás de acuerdo o en desacuerdo, o no lo sabes.

1. **Las dificultades de lubricación (humedad) durante la actividad sexual pueden tener su origen en la ausencia de uno o más periodos menstruales.**

Estoy de acuerdo Estoy en desacuerdo La verdad es que no lo sé

1. **La dispareunia (dolor en la penetración) no puede ser tratada por el fisioterapeuta.**

Estoy de acuerdo Estoy en desacuerdo La verdad es que no lo sé

1. **La dificultad de llegar al orgasmo y el dolor en las relaciones sexuales se pueden mejorar con ejercicios de los músculos del suelo pélvico.**

Estoy de acuerdo Estoy en desacuerdo La verdad es que no lo sé

1. **La realización de actividad física moderada contribuye a prevenir la aparición de síntomas de disfunciones sexuales (por ejemplo, la falta de lubricación).**

Estoy de acuerdo Estoy en desacuerdo La verdad es que no lo sé

1. **La actividad deportiva de alto impacto (salto, carrera, lanzamiento,…) puede estar relacionada con mayor dificultad de llegar al orgasmo.**

Estoy de acuerdo Estoy en desacuerdo La verdad es que no lo sé

1. **No merece la pena acudir al médico para contarle las dificultades para llegar al orgasmo porque no existe ninguna solución médica a estos problemas.**

Estoy de acuerdo Estoy en desacuerdo La verdad es que no lo sé

1. **Las molestias o dolor durante la actividad sexual con penetración vaginal puede estar relacionada con la hiperactividad de músculos del suelo pélvico.**

Estoy de acuerdo Estoy en desacuerdo La verdad es que no lo sé

1. **Tener una buena musculatura del suelo pélvico mejora la satisfacción sexual.**

Estoy de acuerdo Estoy en desacuerdo La verdad es que no lo sé

1. **La sequedad vaginal (falta de lubricación) solo se puede tratar con medicamentos u otros productos farmacológicos aplicados a nivel vaginal.**

Estoy de acuerdo Estoy en desacuerdo La verdad es que no lo sé

1. **El estreñimiento se relaciona con la aparición de molestias o dolor durante las relaciones sexuales.**

Estoy de acuerdo Estoy en desacuerdo La verdad es que no lo sé

**CONOCIMIENTOS SOBRE DISFUNCIONES ANORRECTALES (INCONTINENCIA ANAL)**

A continuación, te presentamos una serie de preguntas sobre la función ano-rectal. Por favor, contesta en cada una de las preguntas si estás de acuerdo o en desacuerdo, o no lo sabes.

1. **La práctica de actividad deportiva de alta intensidad está ligada a mayor probabilidad de incontinencia anal (pérdida involuntaria de gases y/o heces).**

Estoy de acuerdo Estoy en desacuerdo La verdad es que no lo sé

1. **Puede considerarse normal una frecuencia de defecaciones/evacuaciones de 2 veces por semana.**

Estoy de acuerdo Estoy en desacuerdo La verdad es que no lo sé

1. **Una vez que hemos sentido la primera sensación de ganas de defecar, es importante ir a evacuar/defecar de forma inmediata.**

Estoy de acuerdo Estoy en desacuerdo La verdad es que no lo sé

1. **Las heces en forma de bolas puede ser un signo de estreñimiento**

Estoy de acuerdo Estoy en desacuerdo La verdad es que no lo sé

1. **En personas sedentarias, el estreñimiento puede ser mejorado mediante la realización de actividad física.**

Estoy de acuerdo Estoy en desacuerdo La verdad es que no lo sé

1. **El estreñimiento es más frecuente en personas jóvenes que en personas mayores.**

Estoy de acuerdo Estoy en desacuerdo La verdad es que no lo sé

1. **Beber suficientes líquidos (agua) durante el día está relacionado con la prevención del estreñimiento.**

Estoy de acuerdo Estoy en desacuerdo La verdad es que no lo sé

1. **Una vez que se tienen síntomas de escapes de gases y/o heces solo es posible controlarlos con el uso de compresas/salvaslips.**

Estoy de acuerdo Estoy en desacuerdo La verdad es que no lo sé

1. **La realización de ejercicios de suelo pélvico puede ayudar a mejorar el estreñimiento.**

Estoy de acuerdo Estoy en desacuerdo La verdad es que no lo sé

1. **El estreñimiento crónico está relacionado con la aparición de prolapsos de órganos pélvicos.**

Estoy de acuerdo Estoy en desacuerdo La verdad es que no lo sé

**CONOCIMIENTOS SOBRE PROLAPSO DE ÓRGANOS PÉLVICOS**

A continuación, te presentamos una serie de preguntas sobre el prolapso de órganos pélvicos (que supone el descenso (prolapso) de la vagina, útero, vejiga o recto a través de la vagina). Por favor, contesta en cada una de las preguntas si estás de acuerdo o en desacuerdo, o no lo sabes.

1. **El prolapso de órganos pélvicos (descenso de vagina, útero, vejiga o recto) es más frecuente en mujeres jóvenes que en mujeres mayores.**

Estoy de acuerdo Estoy en desacuerdo La verdad es que no lo sé

1. **El prolapso de órganos pélvicos puede aparecer a cualquier edad.**

Estoy de acuerdo Estoy en desacuerdo La verdad es que no lo sé

1. **Ciertos ejercicios pueden ayudar a detener o evitar que el prolapso de órganos pélvicos empeore.**

Estoy de acuerdo Estoy en desacuerdo La verdad es que no lo sé

1. **Los síntomas del prolapso de órganos pélvicos pueden incluir pesadez y/o presión en la pelvis.**

Estoy de acuerdo Estoy en desacuerdo La verdad es que no lo sé

1. **Examinar a la mujer es una buena manera para que un médico diagnostique un prolapso de órganos.**

Estoy de acuerdo Estoy en desacuerdo La verdad es que no lo sé

1. **Una vez que la mujer tiene un prolapso de órganos pélvicos, no se puede hacer mucho para ayudarla.**

Estoy de acuerdo Estoy en desacuerdo La verdad es que no lo sé

1. **Levantar peso a diario puede provocar un prolapso de órganos pélvicos.**

Estoy de acuerdo Estoy en desacuerdo La verdad es que no lo sé

1. **Las personas obesas tienen menos probabilidad de tener un prolapso de órganos pélvicos.**

Estoy de acuerdo Estoy en desacuerdo La verdad es que no lo sé
